# Non-Metabolic Membrane Tubulation and Permeability Induced by Bioactive Peptides

**DOI:** 10.1371/journal.pone.0000201

**Published:** 2007-02-14

**Authors:** Antonin Lamazière, Fabienne Burlina, Claude Wolf, Gérard Chassaing, Germain Trugnan, Jesus Ayala-Sanmartin

**Affiliations:** 1 Institut National de la Santé et de la Recherche Médicale (INSERM), UMR 538, CHU Saint Antoine, Paris, France; 2 Université Pierre et Marie Curie, CHU Saint Antoine, Paris, France; 3 UMR Centre National de la Recherche Scientifique (CNRS) 7613, Université Pierre et Marie Curie, Paris, France; Center for Genomic Regulation, Spain

## Abstract

**Background:**

Basic cell-penetrating peptides are potential vectors for therapeutic molecules and display antimicrobial activity. The peptide-membrane contact is the first step of the sequential processes leading to peptide internalization and cell activity. However, the molecular mechanisms involved in peptide-membrane interaction are not well understood and are frequently controversial. Herein, we compared the membrane activities of six basic peptides with different size, charge density and amphipaticity: Two cell-penetrating peptides (penetratin and R9), three amphipathic peptides and the neuromodulator substance P.

**Methodology/Principal Findings:**

Experiments of X ray diffraction, video-microscopy of giant vesicles, fluorescence spectroscopy, turbidimetry and calcein leakage from large vesicles are reported. Permeability and toxicity experiments were performed on cultured cells. The peptides showed differences in bilayer thickness perturbations, vesicles aggregation and local bending properties which form lipidic tubular structures. These structures invade the vesicle lumen in the absence of exogenous energy.

**Conclusions/Significance:**

We showed that the degree of membrane permeabilization with amphipathic peptides is dependent on both peptide size and hydrophobic nature of the residues. We propose a model for peptide-induced membrane perturbations that explains the differences in peptide membrane activities and suggests the existence of a facilitated “physical endocytosis,” which represents a new pathway for peptide cellular internalization.

## Introduction

Processes such as neuromodulation, toxicity, and cell communication can be regulated by natural peptides and protein basic domains. This group encompass several hormones, and antibacterial peptides. Beside these peptides, many proteins have been identified for their capacity to enter cells and reach the cytoplasm. This includes proteins such as homeoproteins, Tat from HIV or the Anthrax and Cholera toxins. The penetration of these proteins into cells is mediated by the presence of basic domains termed protein transduction domains. This has led to the development of short cell-penetrating peptides (CPPs) which are able to enter eukaryotic cells and carry bioactive molecules (for review see [1]). Some of these CPPs also present antimicrobial properties [2], [3]. Generally, the antimicrobial peptides (AMPs) corresponding to the innate immune system are basic and are able to bind and strongly destabilize membranes of bacteria. On the other hand, some neuromodulator peptides possess a dual interaction mode with cells. For example, Substance P (SP) binds to its specific membrane receptor (NK1) and it also binds lipid monolayers due to its partial penetration in the lipid domain [4]. Moreover, SP has been reported to enter mast cells lacking the NK1 receptor [5]. The study of these peptides activities is currently rising after the recent occurrence of synthetic peptides designed to stimulate, penetrate and eventually kill living cells. The synthetic peptides comprise peptides such as polyarginine, polylysine or model amphipathic peptides (for review see [1]). All of these peptides are potential therapeutic molecules for the delivery of active molecules [6] and drugs.

These peptides (SP, CPPs, AMPs) share a strong basic character and can be classified in two families: amphipathic and non-amphipathic. The amphipathic character is either related to the charge location in the primary sequence as for SP and the CPP Pep-1 [7] or to the secondary structure (α-helix) as for the Model Amphipathic Peptides [8]. Some amphipathic helical peptides are able to form oligomers leading to an increase of the plasma membrane permeability (“pores”). On prokaryotic cells, the permeabilization by AMPs depends on various factors such as peptide length, hydrophobicity and charged/hydrophobic surfaces ratio. Two permeation mechanisms have been proposed so far: i) the barrel-stave model in which the amphipathic peptides aggregate and insert into the lipid bilayer forming a channel-like transmembrane structure. ii) The toroidal model or detergent-like mechanism which is more deleterious to the membrane structure. The peptide interacts with the membrane surface and curves strongly the bilayer so the pore is lined by headgroups associated with peptides. In this case, short peptides not long enough to span the membrane are able to form pores. [9]–[13].

By contrast, basic CPPs such as Tat, R9 or penetratin (pAntp) do not exhibit any lytic activities on mammalian cells. Both AMP and CPP membrane activities are receptor-independent. The studies of fluorescent CPPs on cultured cells have revealed that different endocytic pathways contribute to the internalization of CPPs: clathrin-independent endocytosis [14], the caveolae-mediated endocytosis [15] or macropinocytosis [16]. In neurons, and in contradiction with the endocytic hypothesis, penetratin internalization was found to be energy-independent [17] (for review see [18]).

Penetratin adopts a partially amphipathic α-helical structure at the membrane surface [19], which is shifted to a β-structure when the peptide/lipid ratio increases [20], [21]. The CPPs corresponding to the helix III of different homeoproteins present variable penetration efficiencies (“snorkelling”) in SDS micelles. Paradoxically, the peptide with the highest cellular uptake efficiency was found to be the most superficial in SDS micelles [22]. Penetratin is not internalized in phosphatidylcholine/phosphatidylglycerol (PC/PG) Large Unilamellar Vesicles (LUVs) [23] however, penetration increased in the presence of a transbilayer potential [24]. The efficiencies of translocation depended on the lipid composition. At membrane saturation concentrations Penetratin and R8 provoked a weak leakage of calcein from PC/PG LUVs [25], [26].

The mechanisms of CPPs membrane translocation are still in debate. Many models of CPPs were proposed explaining the cellular uptake: direct membrane penetration, inverted micelle formation [17], penetration by endocytosis [14], or macropinocytosis [16]. The absence of molecular explanations for the role of energy-independent steps in cell uptake results in controversial data [18].

**Figure 1:**
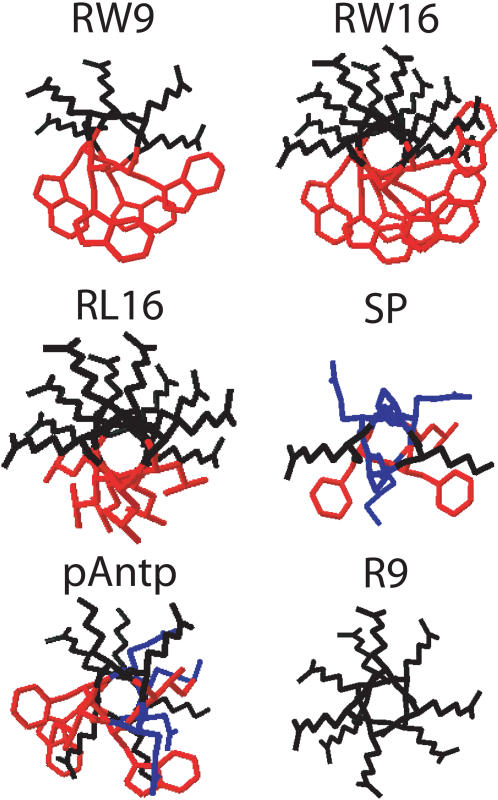
Projections of α–helices of the six basic studied peptides. Basic residues in black, hydrophobic residues in red, other residues in blue. The structure of SP associated to membranes is not known. pAntp is only ∼70% helical when interacting with membranes. The basic/hydrophobic surfaces ratio of RL16 is higher than that of RW16. Helices were generated with the Swiss-PdbViewer programme.

The first step of peptide-cell interaction involves the association of the peptide with the phospholipid components of the outer membrane leaflet. We have selected three different sorts of basic peptides able to bind with negatively charged membranes: the primary amphipathic peptide substance P (SP), the cell-penetrating peptides penetratin (pAntp) [27] and R9 [28] and three secondary amphipathic peptides [29] (table 1 and figure 1). We focus on the relationship between the total charge, length and amphipathicity of these peptides and their effects on membranes. The effects of peptides have been analyzed using small angle X ray diffraction, microscopy of Giant Unilamellar Vesicles (GUVs), experiments of calcein leakage from LUVs, turbidimetry and tryptophan fluorescence. The permeability and the toxicity have been analyzed on cell cultures. Depending on their charge and amphipathic character these peptides induce different effects on membrane bilayers including the formation of tubes and vesicles adhesion and/or the formation of pores. Relationships have been found between membrane aggregation and tube formation and size of membrane pores and the nature of the hydrophobic residues. The deformations of PC/PG bilayers on GUVs leading to tubes and vesicles may be stabilized by the formation of peptides/lipids complexes. These physical “endocytosis-like” deformations may represent a new energy-independent pathway for the cellular uptake of cell penetrating peptides, transcription factors containing protein transduction domains and toxins.

**Table 1:** Sequences and physicochemical features of peptides.

Name	Sequence	Length	Charge^a^	Amphipathic^b^
Penetratin (pAntp)	RQIKIWFQNRRMKWKK-NH_2_	16	7	no
Polyarginine (R9)	RRRRRRRRR-NH_2_	9	10	no
RW9	RRWWRRWRR-NH_2_	9	7	yes
RW16	RRWRRWWRRWWRRWRR-Bi	16	10	yes
RL16	RRLRRLLRRLLRRLRR-Bi	16	10	yes
Substance-P (SP)	RPKPQQFFGLM-NH_2_	11	3	no

a) Net theoretical positive charge at pH 7. (R9, RW9 and SP contained a free N-terminal amino group. RW16 and RL16 contained a biotin (Bi) on N-terminus. Both biotinylated and acetylated penetratin derivatives were also tested in experiments and gave the same results than the non-modified peptide).

b) Amphipathic α-helix when bound to membranes

## Results

### Membrane deformations induced by peptides on giant unilamellar vesicles

To study the peptide-induced mesoscopic deformations of membranes by basic peptides, we prepared giant unilamellar vesicles (GUVs: PC/PG (9/1)). Six peptides were studied. The neuromodulator substance P (SP), five cell-penetrating peptides, two mainly basic: Polyarginine (R9), and Penetratin (pAntp), and three amphipathic: polyarginine-tryptophane (RW16 and RW9) and polyarginine-leucine (RL16) (table 1 and figure 1).

**Figure 2:**
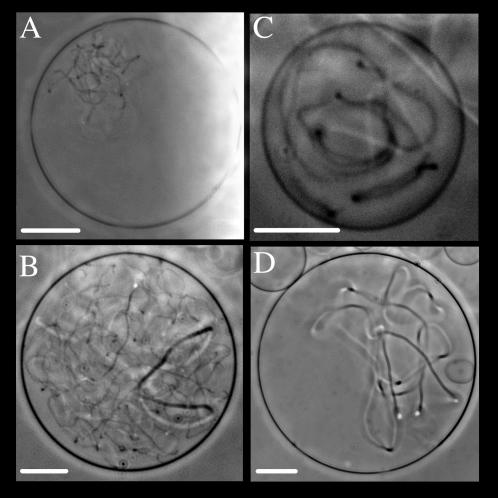
Membrane deformations on GUVs (PC/PG 9/1) induced by two CPPs. Tubular structures observed in vesicles incubated with R9 peptide (A, B), and pAntp (C, D). Phase contrast microscopy at 25°C. Scale bar 20 µm.

The cell penetrating peptides pAntp and R9 showed very similar effects on GUVs. They induced membrane invaginations in the form of micro-tubes that grow inside vesicles (Fig. 2). The tubes are able to move inside the vesicles. The growing micro-tubes invade the entire vesicle internal volume in less than 20 minutes (Video S1). Although we did not quantify tubes, R9 seems to form more numerous tubes than pAntp with a higher “filling” rate (compare panels B versus C, D of figure 2). The evolution of tubes showed that vesicle integrity was maintained and that usually, the larger diameter tubes came from a process of thin tubes enlargement (Video S2). Moreover, these peptides were able to induce the adhesion of adjacent membranes when vesicles were initially in close contact (Video S3).

**Figure 3:**
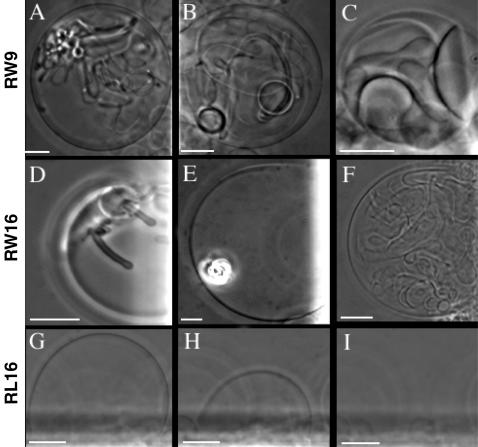
Membrane deformations on GUVs (PC/PG 9/1) induced by amphipathic peptides. Coexistence of tubes and small vesicles inside the GUVs (A, B) and adhesion of GUVs by RW9 (C). Tubes formation (D) membrane aggregates (E) and GUVs adhesion and internal vesicles (F) induced by RW16 peptide. Time-lapse sequence of a GUV burst induced by RL16, t = 0 s (G) t = 2 s (H) and t = 3 s (I). Scale bar 10 µm.

The peptide RW16 exhibited a different behaviour. Indeed, tubes were also observed for RW16 with the same dynamics for growth and mobility than for R9 and pAntp peptides. However, tubes induced by RW16 reach more frequently greater diameter (fig. 3D and Video S4). RW16 is also able to form small lipid aggregates inside the vesicles corresponding to the bright spots detected in Fig 3E. This causes a dramatic perturbation to the vesicle and leads to a gradual reduction of GUV size. RW16 induced also large endosome-like vesicle formation in a single step without detectable preliminary formation of tubes (fig. 3F). The frequency of adhesion of adjacent membranes was higher than with pAntp or R9. Finally, RW16 induced also occasional bursting of the GUV.

The shorter peptide, RW9 showed effects similar to RW16, R9 and pAntp. As shown in fig 3A and 3B, thin and large tubes, small lipid aggregates and endosome-like vesicles coexist in the same GUV with frequent adhesions. GUVs integrity was not altered by the peptide and all the membrane fragments are enclosed moving inside the vesicle. Moreover, as RW16, RW9 also induces vesicle formation (fig. 3F). It was observed that some vesicles were formed by fluctuations at the level of adhering membranes.

Amphipathic peptide RL16 induces complete GUV destruction after few seconds (fig. 3G,H,I). In contrast with other peptides, Substance-P did not perturb GUVs morphology even after 6 hours of incubation at high peptide concentration (50 µl of peptide solution at 0.5 mM in 2 ml of buffer) (not shown). A summary of results is presented in table 2.

**Table 2:** Morphological effects of peptides on giant unilamellar vesicles (GUVs)^a^

Peptides Effects on GUVs	pAntp	R9	RW9	RW16	RL16	SP
Thin tubes	+	++	++	+/−	+/−	−
Large tubes/vesicles	+++	+++++	+++++	++	−	−
Adhesion	+	++	+++	++	−	−
Burst	−	−	+/−	+	++	−

a) The quantification of effects was obtained by observation of 153 recorded GUVs. The number of GUVs containing the different structures and adhering to other GUVs was counted, however the number of tubes or vesicles induced by the peptides was not measured due to the frequent high density of structures inside the vesicles. The evolution of fines tubes to large tubes and vesicles increases the difficulty to quantify precisely the proportion of structures.

Two phenomena were considered for further studies: 1) Adhesion of adjacent membranes (this property may be related to stabilization and modulation of tube diameter) and 2) membrane permeabilization, which would be responsible for GUV bursting.

### Peptides induce changes on membrane lipids organization

**Figure 4:**
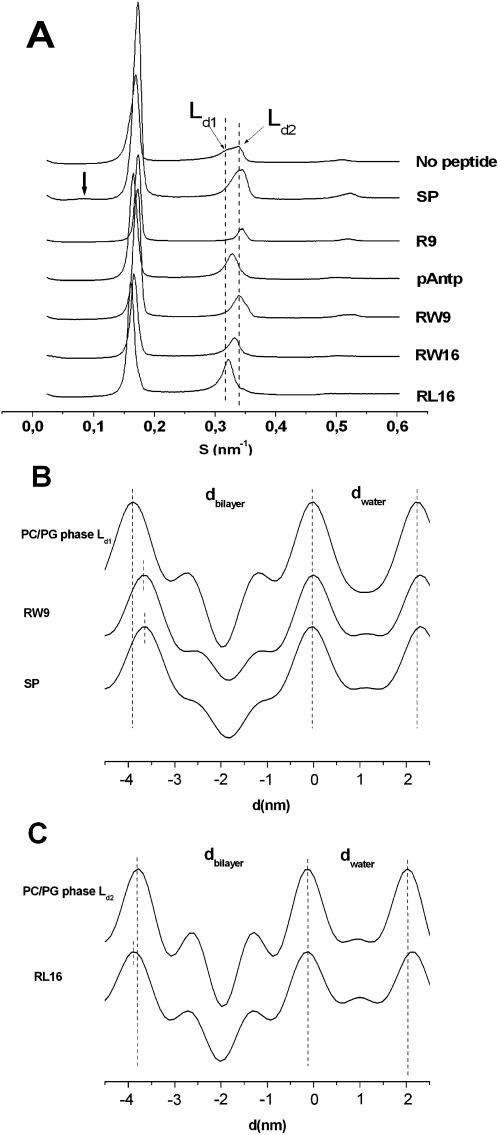
Membrane thickness alteration induced by peptides. A) Diffractograms of PC/PG (9/1) MLVs in the absence or presence of peptides with a weight ratio peptide/lipid of 1/20 (at 20°C). Thick arrow shows the position of a weak non lamellar contribution induced by substance P. Ld1 and Ld2 are two lamellar phases clearly distinguished by slightly different d-spacings. B) Electron density profiles of Ld1 phase of peptide free MLVs or formed in the presence of RW9 and SP peptides. C) Electron density profiles of Ld2 phase of peptide free MLVs or formed in the presence of RW16 and RL16.

Tube formation could be a result of peptide-induced membrane negative curvature. In agreement with this hypothesis, it has been shown that amphipathic peptide orientation is modulated by the lipid capability to favour a positive or negative curvature [30] (i.e. the increase in phosphatidylethanolamine, which induces negative curvature increases the critical amphipathic peptide concentration, needed for pore formation). Moreover, this phenomenon was suggested to be related to bilayer thickness variations but it is still controversial whether amphipathic peptides thin down [31], [32] or thicken [33] membranes. Moreover, as far as we know, effects of basic CPPs on model membranes have not been studied by Small Angle X ray Scattering (SAXS). Therefore, we investigated the effect of the six peptides on the arrangements of the membrane bilayer lipids by SAXS. This technique is adapted to the measurement of the bilayer thickness and to the detection of highly curved non lamellar lipid arrangements such as cubic or hexagonal phases [34]. All samples were fully hydrated in order to avoid any artefactual thinning effects [33]. Figure 4A shows that PC/PG (9/1) multilamellar vesicles present two separated lamellar phases with different d-spacings (Ld1 and Ld2). The temperature dependent behaviour of the samples and the wide-angle scattering (not shown) showed that the two phases are in the liquid disordered state (Ld) [35]. The peptides were co-solubilised with the lipids so the peptide is stacked between the bilayers at a Peptide/Lipid ratio of 1/20 (w/w). The highly cationic peptide R9 inhibited the membrane heterogeneity in favour of a unique liquid disordered phase (fig 4A). The other five peptides kept the heterogeneity. Moreover, except for SP, which showed a weak diffraction corresponding possibly to a non lamellar phase, the other peptides did not induce any transitions to a non lamellar phase within this particular membrane composition (PC/PG; 9/1). In figure 4A, it is clear that the peptides changed the position of the maxima indicating either a modification of the diffraction repeat distances or a modification in the relative contribution of the two phases. Because the diffraction peak intensities reflect not only the amount of the phase-forming lipids but also the form factor we calculated the electron density profiles from the diffractogram of each lamellar phase. As noted in table 3, we observed differences of membrane and water layer thickness in the presence of peptides. Significant thinning was observed on the Ld1 phase for RW9 and SP (Fig 4B). The bilayer thickness was decreased by both peptides (−2.7 and −2.8 Å, respectively). Significant changes were also observed for RL16 on the Ld2 phase (fig 4C). RL16 induced an increase of +1.3 Å of the membrane thickness and +1.7 Å in the hydration layer.

**Table 3:** Effect of the peptides on the thickness of the membrane bilayer (d_bilayer_) and hydration layer (d_water_) of the two distinct lamellar phases (Ld1 and Ld2) comprised in the PC/PG (9/1) multilamellar vesicles at 20°C.

	PC/PG		pAntp		RW9		RW16		RL16		SP	
	L_d1_	L_d2_	L_d1_	L_d2_	L_d1_	L_d2_	L_d1_	L_d2_	L_d1_	L_d2_	L_d1_	L_d2_
S^−1^ (nm)	6.13	5.81	6.06	5.94	5.96	5.74	6.08	5.89	6.20	6.01	5.96	5.75
d_bilayer_ (nm)	3.90	3.62	3.78	3.63	3.63	3.55	3.83	3.71	3.93	3.75	3.62	3.54
d_water_ (nm)	2.23	2.19	2.28	2.31	2.33	2.19	2.25	2.18	2.27	2.26	2.34	2.21

### Peptides induce membrane adhesion and vesicles aggregation

**Figure 5:**
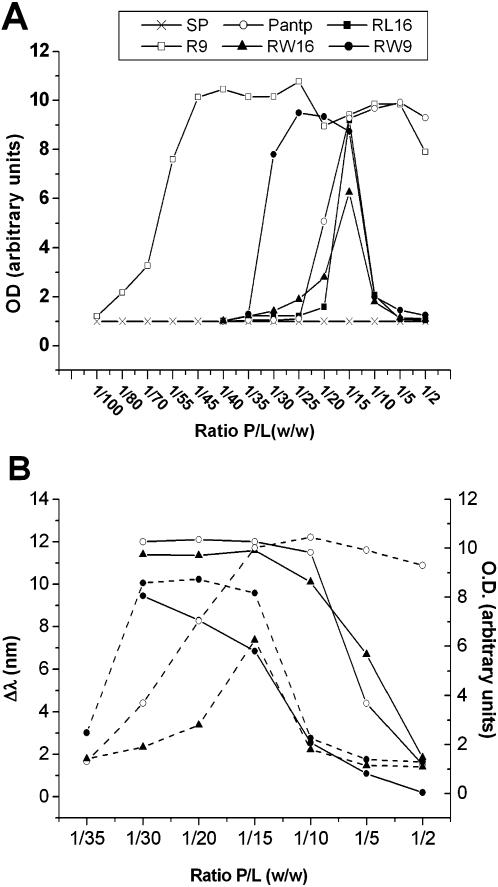
Membrane bridging capacity of peptides. A) Aggregation profiles of PC/PG (9/1) vesicles at different Peptide/Lipids weight ratios: R9 (○), pAntp (□), RW9 (•), RW16 (▪), RL16 (▴) and SP (X). Optical density (OD) was recorded at the plateau of aggregation (20 min after peptide addition). B) Membrane binding and aggregation of RW16, RW9 and pAntp peptides. Shift in emission wavelength (λ shift) in function of P/L ratio (solid lines). LUVs aggregation (dotted lines). For presentation facility, data were adjusted in arbitrary units. pAntp (□), RW9 (•), RW16 (▪). All experiments were achieved at 25°C.

In order to quantify peptides ability to provoke membranes adhesion we measured the aggregation of PC/PG (9/1) large unilamellar vesicles (60–100 nm LUVs) by monitoring the turbidity of the sample. As shown in fig 5A, Substance-P that showed no effect on GUVs does not aggregate LUVs. R9 and pAntp show similar aggregation profiles consisting of an increase of aggregation to reach a plateau value. R9 started to aggregate LUVs at a peptide/lipid mass ratio (P/L) of 1/80 and reached the plateau at 1/45. pAntp needs higher peptide concentrations and started to aggregate LUVs at P/L ratio of 1/25 with a plateau at 1/15.

Amphipathic peptides RW9, RW16 and RL16 exhibit a peak-like profile. The small peptide RW9 showed a large peak for aggregation starting at P/L 1/35 followed by a decrease at P/L of 1/10. RW16 and RL16 showed sharper peaks starting LUV aggregation at P/L ratio around 1/25 and a decrease at P/L ratio of 1/10.

To study these differences between the amphipathic and non-amphipathic peptides, we analyzed the changes in tryptophan fluorescence of pAntp, RW16 and RW9 at different P/L ratios. When tryptophan residues move from a polar to a less polar environment, the fluorescence emission shifts to lower wavelength indicating lipid binding (from 356 nm to 344 nm for our peptides) (see also [36]). In figure 5B, we show that the three peptides are completely bound to membranes at low P/L ratio. The saturation for pAntp and RW16 was found at a P/L ratio of 1/10, and for RW9 at 1/15. The maximal shift in wavelength (peptide-membrane saturation) correlated with maximal aggregation. At higher P/L ratios, the wavelength shift decrease indicating the presence of non-bound peptide. At saturation, pAntp did not change its capacity to aggregate LUVs but, on the contrary, for the amphipathic peptides RW16 and RW9, saturation of the membranes with the peptides blocked LUV aggregation (Fig 5B). This was interpreted as a change in peptide organisation at the membrane surface that results in peptide arrangement competent for pore forming and non-competent with aggregation (see discussion).

### Membrane permeability and cell toxicity

**Figure 6:**
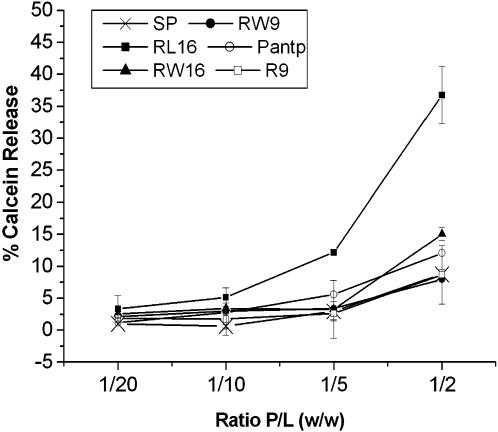
Peptide-induced LUVs permeability at different Peptide/Lipid weight ratio. Percent of calcein release from LUVs 5 min after peptide addition: R9 (○), pAntp (□), RW9 (•), RW16 (▪), RL16 (▴) and SP (X). Experiments were achieved at 25°C.

The size reduction and collapse of GUVs incubated with RL16 and to a lower extent with RW16 suggested the capability of amphipathic peptides to permeabilize membranes. Permeability was first followed by calcein release from LUVs. Permeabilization of the membrane results in calcein release, dilution, and fluorescence increase. As shown in figure 6 and table 4, only peptide RL16 was able to induce a significant calcein release from LUVs at a P/L ratio of 1/5.

**Table 4:** Membrane permeabilization and cell toxicity of peptides.

	pAntp	R9	RW9	RW16	RL16	SP
Calcein release^a^	11%	8%	7%	14%	38%	8%
Cell permeabilization^b^	no	no	no	yes	yes	no
Lethality^c^	0	0	0	0	55%	0

a) Percent of calcein released from LUVs at a peptide/lipid ratio of 1/2

b) Monitored by calcium permeability inducing annexin 2 movement in MDCK cells

c) After 48 hours of incubation of CHO cells with 10 µM peptides

**Figure 7:**
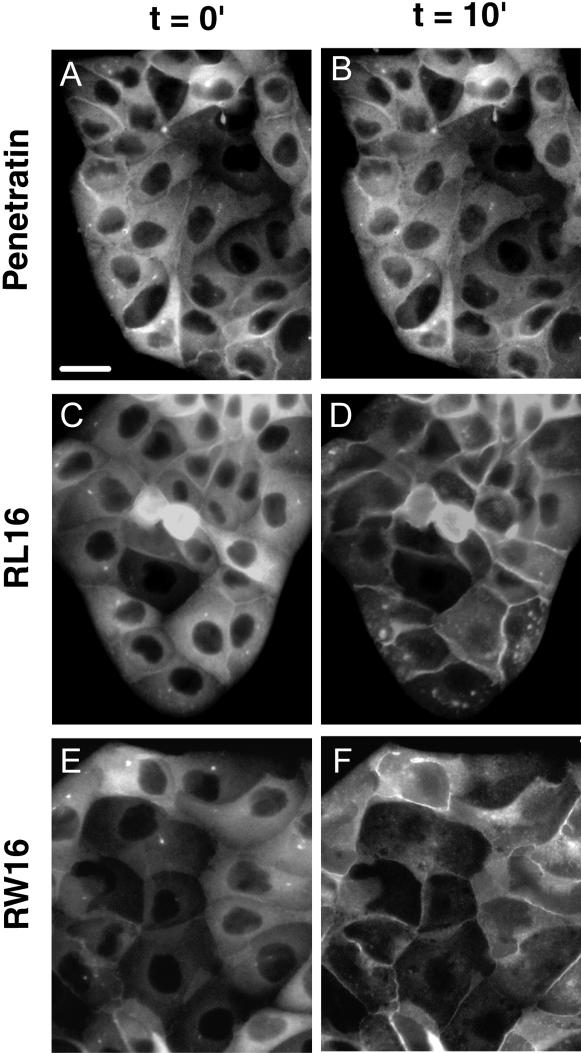
Movement of Annexin 2-GFP by peptide-induced ion permeabilization of the plasma membrane. Visualisation by fluorescence microscopy of the Anx2-GFP migration from the cytsol to the plasma membrane before and after addition of peptides (10 µM). pAntp, RL16 and RW16. (A,C,E before peptide addition, B,D,F 10 min after peptide addition). Scale bar 10 µm.

Cell permeability was also analyzed in Annexin 2-GFP transfected MDCK cells. Annexin 2 is a Ca^2+^-dependent membrane binding protein. We took advantage of this property to observe the rise of intracellular Ca^2+^ concentration provoked by the influx of ions through the peptide-permeabilized plasma membrane by monitoring the fluorescent GFP-protein binding to the plasma membrane. Cells were incubated with different peptide concentrations. pAntp, SP, R9 and the short amphipathic peptide RW9 did not induce any increase in permeability towards ions even at high peptide concentration (100 µM) (fig 7B and data not shown). By contrast, the two long amphipathic peptides RW16 and RL16 (10 µM) provoked the Ca^2+^ influx and the subsequent mobilization of annexin 2 from cytosol to the plasma membrane in 10 minutes (fig 7D, 7F and Video S5). In absence of extracellular Ca^2+^ no Annexin 2-GFP movement was detected (not shown). The comparison of the behaviour of RL16 and RW16 in calcein leakage from LUVs and Ca^2+^ permeability in cells indicates that the “pores” formed by both peptides are different.

Excessive permeabilization or other membrane perturbations could induce cell toxicity. We therefore measured cell toxicity on CHO cells after 48 hours of peptide incubation. As shown in table 4, the only toxic peptide was RL16.

## Discussion

The characterization of peptides-membranes interactions is fundamental to understand the pathways of peptide internalization and their effect on membrane permeability. In this work, we compared six different peptides using complementary approaches in model membranes and living cells. The use of liposomes is adapted to study the influence of physico-chemical parameters such as lipid composition or membrane curvature and living cells allow an approach with a biological complex membrane. The six studied peptides showed different behaviour on giant vesicles. We have previously reported that the primary amphipathic peptide SP interacts strongly with phospholipids organized either in phosphatidylcholine/phosphatidylserine monolayers or small unilamellar vesicles (SUV) [4]. The electron density profiles of small angle X ray diffraction of the PC/PG lamellar phases performed in this study also showed a strong peptide-lipid interaction. Indeed, SP and RW9 led to the most important decrease of the bilayer thickness. However, SP showed no effect in all other experiments performed suggesting that it remains inserted in PC/PG LUV and GUV bilayers without disturbing the local supra-molecular organization of lipids. This is probably related to the low number of positive charges of the peptide (only 3 positive charges within 11 residues) and to shape complementarities between the peptide and the phospholipids.

**Figure 8:**
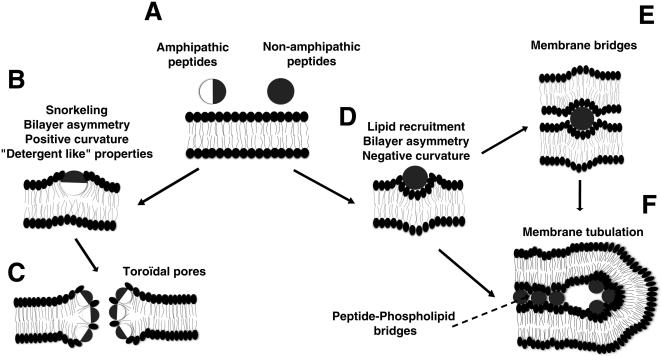
Model for peptide-membrane interaction. A) Two main properties are considered: amphipathicity and net positive charge. Changes in these properties result in different degrees of membrane perturbations. Basic surface in dark, hydrophobic surface in white. B) The binding of the amphipathic peptides involves electrostatic interactions between the basic residues and lipid headgroup negative charges, and hydrophobic interactions with lipid fatty acyl moieties. A strong snorkelling effect of the peptide could induce protrusion of headgroups attracted by the charged helix residues inducing lipid reorganisation resulting in asymmetry of the bilayer halves and positive curvature of the membrane. C) The detergent property of amphipathic peptides and the induced positive curvature result in the formation of toroidal pores. D) The binding of basic peptides mainly involves electrostatic interactions with the lipid headgroup phosphates. This results in the recruitment of phospholipids and then in membrane asymmetry with negative curvature. E) The bridging interaction of the peptide between two membranes allows adhesion and vesicles aggregation. F) Membrane tubulation results from the membrane curvature inducing invaginations. The thin tubes could be stabilized by the bridging properties of the peptides between membranes apposed faces.

The other peptides induced different types of effects on PC/PG liposomes. Membrane tubulation, adhesion, vesiculation or bursting were observed with GUVs. The peptides also caused LUV aggregation or permeabilization. The behaviour of these peptides bearing at least seven positive charges could be explained by the different models of lipid-peptide interaction presented in figure 8, which include the previously reported toroidal pore model [9]. The different effects observed on the membrane seemed to be related to the peptide degree of amphipathicity. For simplification, only the two limit classes of basic non amphipathic and amphipathic peptides were considered in figure 8.

The amphipathic peptide RL16 may bind membranes by two types of interactions: the electrostatic interaction of basic residues with negatively charged phosphate groups of phospholipids and by hydrophobic residues with the fatty acyl chains of the bilayer. If this “snorkelling” effect is strong and able to induce a lipid rearrangement between the two membrane leaflets, the peptides allow positive curvature on the membrane (fig 8B). This model is similar to that suggested by Mangavel [37] for the amphipathic peptide KL20. This peptide lies parallel to the plane of the bilayer of Small Unilamellar Vesicles. The authors suggest that the phospholipid headgroups protrude from the bilayer towards the solvent due to electrostatic interactions with the amino groups of the peptide. This favours membrane aggregation and modifies membrane thickness. We showed that RL16 induces a significative increase of the bilayer thickness. This increase may be related to the protrusion suggested for the phospholipid headgroups and may be important for membrane permeabilization. Two models of permeation mechanisms have already been proposed: i) the barrel-stave model in which the amphipathic peptides aggregate and insert into the lipid bilayer with the hydrophobic amino acid residues intercalated between the lipids and the hydrophilic faces forming the inside wall of the pore. In this model, the peptide is long enough to span the bilayer. ii) The toroidal model or detergent-like mechanism in which the peptide interacts with phospholipid headgroups on the membrane surface and curves strongly the bilayer so the pore is lined by headgroups associated with peptides. In this case, short peptides not long enough to span the membrane are able to form lipoproteic pores. [9]–[13]. RL16, which induces formation of “pores” is able to release widely calcein from LUVs, provokes massive GUVs burst, permeabilize and kill cells. In contrast, RW16 seems to form smaller pores since it is able to induce calcium permeabilization of cell membranes and occasional GUV burst but does not induce calcein release from LUVs. RL16 increases membrane thickness by 1.3 Å and RW16 by only 0.9 Å. For RL16 and RW16 peptides, “solvation” could generate transient effects on the membrane structure leading to toroidal-like pores (fig 8C). Both peptides must be able to recruit phospholipids differently depending on their charges to optimise shape complementarities. In support of the proposed membrane positive curvature induced by amphipathic peptides, it was shown that lysoPC, a lipid with positive curvature tendency, facilitate the formation of toroidal pores by alameticin and melitin [10], [30]. RL16 induced essentially the bursting of giant vesicles. This result is similar to a previous study showing that “short” amphipathic antimicrobial sequences, citropein and aurein (16 and 15 residues respectively) induced the complete and immediate destruction of GUVs [38]. Like citropein and aurein, RL16 is not long enough to span the membrane. Therefore, the detergent-like mechanism is a simple assumption to explain GUVs destruction. However, it does not seem only related to peptide length, hydrophobicity and basic surface extension ratio appear to be crucial since RW16 with the same length does not cause such dramatic effect on GUVs. The hydrophobic indexes decrease from Leu (3.8), Trp (−0.9) to Arg (−4.5) [39] suggesting that the average orientations of peptides must differ between RL16 and RW16. Tryptophans are generally located at the water membrane interface. In summary, its permeabilization power is smaller than that of the RL16 peptide. The explanation is based on the weaker detergency power of the peptide due to the more symmetric distribution of charged and hydrophobic surfaces of the α-helix of RW16 compared to RL16 (fig 1).

For the basic non-amphipathic peptides, where the positive charges are not segregated on a particular area of the helix circumference, membrane binding must be essentially due to electrostatic interactions of basic residues with phosphates. In our model, the recruitment of phospholipids without important snorkelling and low or no phospholipid protrusion would result in negative curvature-competent membrane asymmetry (fig 8D). This binding could also be responsible for membrane aggregation by peptides bridging simultaneously two membranes (fig 8E). The presented X ray diffraction data indicated that SP and RW9 reduced the bilayer thickness. Since SP showed no effects in our experiments and R9 and pAntp showed slight changes of bilayer thickness, we assume that this membrane perturbation is not sufficient to explain tubes formation or membrane aggregation. The basic residues in the surface must be in the right positions (i.e. in all directions) to induce membrane aggregation.

The LUV aggregation curves indicate that the aggregated membranes are quite stable for the non-amphipathic R9 and pAntp peptides. In contrast, the amphipathic peptides showed blockage of aggregation when increasing the P/L ratio. The correlation between the experiments of turbidity and fluorescence maxima shift obtained with RW9 and RW16 peptides indicates that when these peptides reach the surface saturating concentration, they change their conformation and/or peptide-peptide interactions. At this stage, the peptides may not induce aggregation but provoke “pore” formation. This is in agreement with Huang's group results showing that when helical peptides reach a threshold concentration (P/L*) they switch from a parallel to a perpendicular orientation to the plane of the bilayer, this induces formation of pores [10]. This last hypothesis is supported by the fact that RL16 peptide induces calcein leakage from LUVs at the same P/L ratio at which it blocks LUV aggregation. On the contrary, for pAntp peptide the increase of P/L ratio after membrane saturation results in a very low decrease of aggregation suggesting a more stable peptide-lipid interaction. This low decrease can be explained by the conformational change of membrane bound pAntp. At low peptide/lipid ratio pAntp adopts a partially α-helical structure [19], at high peptide/lipid ratio and high PG content the α-helix shifts to a β-structure [20], [21], [40]–[42]. As stated by Persson [43], it is difficult to know whether aggregation induces the pAntp conformational transition from α–helix to β–sheet or vice versa. Based on experiments with PEG-conjugated lipids the authors found that the transition is driven by membrane aggregation, which is consistent with our model in which the α–helix induces the membrane adhesion. A further conformational change (β–sheet transition) could explain membrane breaking apart and tube enlargement for pAntp.

Considering the tubes formation by non amphipathic peptides, and in agreement with our data, it was reported that polylysine induces the formation of membrane tubes in GUVs [44]. Tubes formation could be a consequence of the lipid asymmetry induced by the peptides (fig 8F). The starting driving force for tubulation would be based on the phospholipid asymmetry between inner and outer layers which generates invagination [45], [46]. In model membranes, the two leaflets are symmetric and therefore the surface tension is nil and no budding should occur. Our hypothesis is that highly positively charged CPPs such as R9 or pAntp recruit lipids on the outer monolayer. This process generates a local asymmetry so that the outer layer is compressed and the inner is relatively dilated. In agreement with GUVs experiments, the model predicts that the invagination grows in the form of tubes inside the vesicles. This driving force would be stabilized further by the capability of peptides to bridge membranes. This results in the formation of thin tubes observed for R9, pAntp and RW9. As proposed by Gonçalves et al. [47], membrane adhesion could also be powered by intervesicle peptide bridges. Thus, the membrane bridging property of peptides could stabilize tube growth. It is interesting to note that the R9 and RW9 tubes often remain thin whereas other peptide-induced tubes become larger more frequently. Moreover, it is difficult to explain all these effects without cooperativity between peptides. The deformation of the membrane bilayer and the tubular growth would need the clusterisation of peptides on the membrane surface. The clusterisation would be obtained by phospholipids bridging basic residues of adjacent peptides (Fig. 8F). This property is apparently not related to the amphipathicity of the peptide and seems to be more related to the high density of positive charges present in the peptides. LUV's aggregation is correlated with the GUVs tubulation. Peptides efficient for tubulation such as R9, RW9 and pAntp are also able to aggregate LUVs efficiently at the lower Peptide/Lipid ratios (1/50, 1/30 and 1/20 respectively, figure 5).

In conclusion, we have established that a combination of the number of basic residues and their relative position is important for membrane perturbations. The non-amphipathic basic peptides are able to induce membrane aggregation and tube formation but do not form pores and show low cell toxicity. The amphipathic peptides are able to form tubes and bridges and permeabilize membranes (pores). However, the properties of the amphipathic peptides are widely modulated by their length (the short peptide RW9 seems not to form “pores”) and the distribution of charged versus hydrophobic residues at the surface of the helix gives to the peptides different degree of detergent character and then different pore forming efficiency.

The model of lipid-peptide interaction illustrated in figure 8 sketches the behaviour of two archetypal peptides. pAntp, which does not adopt a perfect amphipathic helix, has an intermediate behaviour and seems to associate to membranes with a certain degree of snorkelling [48].

The different degree of toxicity of amphipatic peptides may be explained by different toroidal pore sizes as showed by the differences in the toxicity of RW16 and RL16. Many models were proposed to explain CPPs cellular uptake: direct membrane penetration, inverted micelle formation [17], or different endocytotic pathways [14], [16]. The absence of molecular explanations for the energy-independent steps in the cell uptake results in controversial data [18]. For the cell penetrating peptides, tube formation could be another important pathway for penetration into cells. Interestingly, the formation of tubular structures can account for the metabolic energy-independent internalization of peptides, which has been a subject of debate. In fact, this capacity of basic domains to invaginate membranes in a “physical” endocytosis-like way would be one of the first steps of cell internalization before the final translocation to the cytosol of basic penetrating peptides. It will be interesting to explore the possibility of this mechanism as a new and general pathway of internalization of proteins such as the proteins with transduction domains, homeoproteins, Tat and toxins.

## Materials and Methods

### Materials

Egg yolk L-α-Phosphatidylcholine (PC), egg yolk L-α-Phosphatidyl-DL-glycerol (PG) and calcein were purchased from Sigma. l-3-Phosphatidylcholine,1-palmitoyl-2-[1-^14^C]palmitoyl ([^14^C] PC ) was obtained from Amersham. Standard *tertio*butyloxycarbonyl (Boc) amino acids, p-methylbenzhydrylamine-polystyrene resin, and O-(benzotriazol-1-yl)-1,1,3,3-tetramethyluronium hexafluorophosphate (HBTU) were purchased from Senn Chemicals (Dielsdorf, Switzerland). Solvents (peptide synthesis grade) and other reagents for peptide synthesis were obtained from Applied Biosystems.

### Peptide Synthesis

Peptides were assembled by solid-phase synthesis on an ABI Model 433A peptide synthesizer (Applied Biosystems) using a standard Boc strategy (amino acid activation with dicyclohexylcarbodiimide/1-hydroxybenzotriazole or HBTU). Peptides were cleaved from the resin by treatment with anhydrous HF (1 h, 0°C) in the presence of anisole (1.5 ml/g peptidyl resin) and dimethyl sulfide (0.25 ml/g peptidyl resin). Peptides were purified by preparative reverse-phase HPLC on a C8 column, using a linear acetonitrile gradient in an aqueous solution of 0.1% (v/v) trifluoroacetic acid. Peptides were >95% pure as assessed by analytical HPLC. Peptide identity was checked by MALDI-TOF mass spectrometry (Voyager Elite, PerSeptive Biosystems) using cyano-4-hydroxycinnamic acid matrix.

### Preparation and visualisation of giant vesicles

Giant unilamellar vesicles (GUVs) were obtained by electroformation as described in [49]. Briefly, 1.5 µl of PC/PG (9/1, w/w) solution (0.4 mg/ml in chloroform/methanol 9/1) were spread on each platinum electrode. The lipid film was dried for half an hour under a gentle stream of nitrogen. The chamber was placed in an inverted microscope (Zeiss Axiovert 200M) and a thermocouple positioned to monitor the temperature (25°C). The electrodes were then completely hydrated with 2 ml of a low ionic strength buffer (HEPES 0.5 mM, pH 7.4 and conductivity 20 µS/cm). Immediately after buffer addition, a low frequency alternating field (5 Hz and 1 V) was applied on the electrodes for at least 2 hours. GUVs of diameter between 10 to 100 µm were observed. 2.5 µl of 50 µM peptides solution were added close to the electrodes (2 ml of buffer) and images were captured with a CCD camera (Cool SNAP HQ) controlled with Metamorph software (Roper Scientific). The observation temperature was 25°C.

### Preparation of LUVs and calcein-loaded LUVs

Large unilamellar vesicles (LUV) were prepared by extrusion through a polycarbonate filter (pore diameter 100 nm) in an extrusion device from Avestin as described in [38]. To obtain calcein loaded LUVs, at 2 mg/ml lipid concentration (PC/PG/ [^14^C] PC 9/1/0.01 mol ratio), a solution in chloroform was dried under a gentle stream of nitrogen and maintained under vacuum for at least 2 h to ascertain removal of residual solvent. Then, the dried lipid film was hydrated with 1 ml of 70 mM calcein solution in HEPES 10 mM pH 7.4, vigorously vortexed and extruded. Free calcein was separated by passing the suspension trough two gel filtration columns in tandem (Econo-Pac 10 DG, Bio-Rad). The final lipid concentration was quantified measuring the [^14^C] PC radiactivity with a liquid scintillation counter LS 6000 SC (Beckman Coulter France).

### Membrane aggregation

LUVs aggregation was monitored by turbidimetry (absorbance at 340 nm) in a Cary spectrophotometer (Varian) as described [50]. Peptides were added to a 500 µl quartz cuvette containing 10 µg of LUVs in a HEPES 10 mM pH 7.4 buffer and the absorbance was followed during 20 min after peptide exposure. At this time, membrane aggregation attained the plateau.

### Measure of calcein leakage from LUVs

The release of calcein after 5 minutes of peptide exposure to LUVs was measured by spectrofluorimetry (Varian eclipse) at excitation and emission wavelengths of 490 nm and 500–650 nm, respectively. The spontaneous leakage of calcein in absence of peptide was found to be negligible. Leakage was expressed as a percent relative of the total amount of dye released by LUVs lysis with 1% Triton X-100, according to equation: % release = 100×(F(t)−F_0_)/(F_T_−F_0_). F(t) is the fluorescence intensity at time t, F_0_ is the fluorescence intensity before peptide addition and F_T_ the fluorescence intensity after LUVs lysis.

### Tryptophan fluorescence

Tryptophan fluorescence was measured following the recommendations described in [51]. Excitation wavelength was 280 nm, and emission spectrum was collected (290–600 nm). Excitation and emission slits were 6 nm and 5 nm respectively. A cross-oriented configuration of the polarizer (Ex_pol_ = 90°, Em_pol_ = 0°) was used in order to reduce LUVs diffusion artefacts.

### X-ray diffraction

Small-angle X-ray scattering (SAXS) measurements were performed at stations 8.2 and 16.1 of the Daresbury Synchrotron Radiation Laboratory (UK) following a protocol adapted from Tessier et al. [52]. Briefly, samples for SAXS examination were prepared by dissolving dry lipids in chloroform/methanol (2/1, vol/vol) and mixing to obtain the indicated proportions. The solvent was subsequently evaporated under a stream of oxygen-free dry nitrogen at 45°C and traces of solvent removed by a storage under high vacuum for 2 days. In order to obtain the Multiple Lamellar Vesicles (MLVs) the dry lipids were hydrated with an equal weight of buffer (5 mM HEPES pH 7.4). The aqueous lipid dispersion was thoroughly stirred to obtain a homogeneous dispersion, sealed under argon and kept until examination at 4°C. For X-ray measurements, lipid samples (∼20 µl) were deposited between two thin mica windows and mounted on a programmable thermal stage (Linkam, UK). The temperature was monitored by a thermocouple (Quad Service, Poissy, France) inserted directly into the lipid dispersion. Samples were exposed for 30 seconds to the beam (1.5 Å wavelength). The SAXS quadrant detector response was corrected for the uneven channel response using a static radioactive iron and S was calibrated for d-spacing using hydrated rat tail collagen. Information on the setup, calibration, and facilities are available on the station web site (http://www.srs.dl.ac.uk/ncd/station82/description.html).

The electron density profiles of lipid bilayers were obtained by Fourier synthesis of x-ray diffraction patterns [53]. Integrated densities were derived from five diffraction orders (estimated spatial resolution <0.12 nm) by measuring the area under each diffraction peak separated and integrated using the software Peakfit (Seasolve).

### Cell permeability and cell toxicity assays

Annexin 2-GFP expression vector was a kind gift of Dr Stephen Moss (London UK). The annexin2-GFP transfected MDCK cloning will be published elsewhere. MDCK cells were cultured as described [54]. Peptides were added at different concentrations and the annexin-GFP fluorescence was followed in a Zeiss inverted microscope. Films were acquired with Metamorph software. Cytotoxicity in CHO cells was evaluated using the CCK8 cell counting kit (Dojindo Laboratories). Briefly, cell suspension (100 µl, 1500 CHO cells per well) were seeded in DMEM plus 10 % FCS in 96-well microtiter plate at 37°C. 10 µl of peptides were added twice to the cells after 24 and 48 hours of cell culture to obtain concentrations of 1, 10, 20 and 50 µM and the toxicity was evaluated 48 after treatment.

## Supporting Information

Video S1 Fine tubes formed in GUVs by R9 peptide.
(1.06 MB MOV)

Video S2 Start of a growing tube induced by pAntp.
(1.62 MB MOV)

Video S3 pAntp induced adhesion of GUVs.
(0.19 MB MOV)

Video S4 Growing tubes induced by RW16 peptide.
(0.99 MB MOV)

Video S5 Annexin 2-GFP movement from cytosol to the plasma membrane induced by
(0.42 MB MOV)
